# Effectiveness of Protease Inhibitor/Nucleos(t)ide Reverse Transcriptase Inhibitor–Based Second-line Antiretroviral Therapy for the Treatment of Human Immunodeficiency Virus Type 1 Infection in Sub-Saharan Africa: A Systematic Review and Meta-analysis

**DOI:** 10.1093/cid/cix1108

**Published:** 2017-12-20

**Authors:** Alexander J Stockdale, Matthew J Saunders, Mark A Boyd, Laura J Bonnett, Victoria Johnston, Gilles Wandeler, Annelot F Schoffelen, Laura Ciaffi, Kristen Stafford, Ann C Collier, Nicholas I Paton, Anna Maria Geretti

**Affiliations:** 1 Malawi-Liverpool-Wellcome Trust Clinical Research Programme, Blantyre; 2 Institute of Infection and Global Health, University of Liverpool; 3 Section of Infectious Diseases and Immunity and Wellcome Trust–Imperial College Centre for Global Health Research, Imperial College London, United Kingdom; 4 Kirby Institute for Infection and Immunity, University of New South Wales, Sydney; 5 Lyell McEwin Hospital, University of Adelaide, South Australia, Australia; 6 Department of Biostatistics, University of Liverpool; 7 London School of Hygiene and Tropical Medicine, United Kingdom; 8 Institute of Social and Preventative Medicine, University of Bern; 9 Department of Infectious Diseases, Bern University Hospital, Switzerland; 10 Department of Infectious Diseases, University Medical Centre Utrecht, The Netherlands; 11 Unité Mixte de Recherche de l’Institut de Rech (UMI), Institute de Recherche pour le Développement, Institute National de la Santé et de la Recherche Medicale, University of Montpellier, France; 12 Institute of Human Virology, University of Maryland School of Medicine, Baltimore; 13 University of Washington School of Medicine, Seattle; 14 Yong Loo Lin School of Medicine, National University of Singapore

**Keywords:** HIV, second-line antiretroviral therapy, protease inhibitor, sub-Saharan Africa, drug resistance

## Abstract

**Background:**

In sub-Saharan Africa, 25.5 million people are living with human immunodeficiency virus (HIV), representing 70% of the global total. The need for second-line antiretroviral therapy (ART) is projected to increase in the next decade in keeping with the expansion of treatment provision. Outcome data are required to inform policy.

**Methods:**

We performed a systematic review and meta-analysis of studies reporting the virological outcomes of protease inhibitor (PI)-based second-line ART in sub-Saharan Africa. The primary outcome was virological suppression (HIV-1 RNA <400 copies/mL) after 48 and 96 weeks of treatment. The secondary outcome was the proportion of patients with PI resistance. Pooled aggregate data were analyzed using a DerSimonian-Laird random effects model.

**Results:**

By intention-to-treat analysis, virological suppression occurred in 69.3% (95% confidence interval [CI], 58.2%–79.3%) of patients at week 48 (4558 participants, 14 studies), and in 61.5% (95% CI, 47.2%–74.9%) at week 96 (2145 participants, 8 studies). Preexisting resistance to nucleos(t)ide reverse transcriptase inhibitors (NRTIs) increased the likelihood of virological suppression. Major protease resistance mutations occurred in a median of 17% (interquartile range, 0–25%) of the virological failure population and increased with duration of second-line ART.

**Conclusions:**

One-third of patients receiving PI-based second-line ART with continued NRTI use in sub-Saharan Africa did not achieve virological suppression, although among viremic patients, protease resistance was infrequent. Significant challenges remain in implementation of viral load monitoring. Optimizing definitions and strategies for management of second-line ART failure is a research priority.

**Prospero Registration:**

CRD42016048985.

## Teaser

In sub-Saharan Africa, second-line ritonavir-boosted protease inhibitor–based antiretroviral therapy led to virological suppression in 69.3% of participants at week 48 and 61.5% at week 96, based on an intention-to-treat meta-analysis of 4558 participants (14 studies) and 2145 participants (8 studies), respectively.

The number of people receiving antiretroviral therapy (ART) in sub-Saharan Africa increased from 7.5 million in 2010 to 17 million in 2015 [1], and expanded treatment access has led to substantial gains in life expectancy [2]. The Joint United Nations Programme on HIV/AIDS (UNAIDS) aspires to further, fast-tracked improvements, with a target for 90% of patients knowing their human immunodeficiency virus (HIV) status, 90% being on ART, and 90% showing virological suppression by 2020 [1]. The World Health Organization (WHO) has advocated a public health approach to HIV control in sub-Saharan Africa, centered on standardized regimens for first-line and second-line therapy and, since 2015, on prompt ART initiation regardless of CD4 cell counts [3]. Recommended first-line regimens comprise 2 nucleos(t)ide reverse transcriptase inhibitors (NRTIs), such as tenofovir disoproxil fumarate (TDF) and lamivudine (3TC), and a nonnucleoside reverse transcriptase inhibitor (NNRTI), principally efavirenz [3]. Current recommended second-line regimens include 2 NRTIs such as zidovudine with 3TC, and a boosted protease inhibitor (PI), with lopinavir/ritonavir (LPV/r) or atazanavir/ritonavir preferred. A recent network meta-analysis has highlighted the current lack of evidence for alternative second-line regimens other than LPV/r with raltegravir [4]. As NRTIs are continued in second-line ART, NRTI resistance acquired during first-line ART might represent an important determinant of efficacy [5, 6].

In 2013, WHO recommended adoption of plasma viral load (VL) monitoring to enable early identification of treatment failure and appropriately guide treatment changes [3]. The level of implementation varies across the region, and even in settings with access to routine VL testing, delays in switching to second-line ART are common [7]. With further expansion in ART use, an increasing number of people in sub-Saharan Africa are at risk of treatment failure and drug resistance [8].

To inform policy related to treatment selection, monitoring, patient management, and access to third-line therapy, systematically collated data on outcomes of second-line ART, impact of prior NRTI resistance, and risk of emergent protease resistance are needed. The aim of this study was to provide a comprehensive overview of data on effectiveness of second-line ART in sub-Saharan Africa and to present pooled estimates of virological and resistance outcomes.

## METHODS

### Search Strategy and Selection Criteria

PubMed, Embase, the Cochrane Register of Controlled Trials, Scopus, and Web of Science were searched for articles published from 1 January 1996 to 28 July 2017 according to a predefined strategy (Supplementary Table 1). References cited in the selected articles and abstracts from the International AIDS Society Conference (2014–2016) and the Conference on Retroviruses and Opportunistic Infections (2014–2016) were also reviewed. We contacted the authors of 15 studies to clarify definitions, obtain additional data, and remove duplications.

#### Types of Studies

We included randomized controlled trials (RCTs) and observational studies that reported the outcomes of second-line ART in sub-Saharan Africa with VL measured at least annually. We excluded studies with <20 participants, to avoid small-sample-size bias, and participants outside sub-Saharan Africa in international trials. We excluded studies without defined criteria for switching to second-line ART. For studies reporting the prevalence of drug resistance at second-line ART failure, we required that an unbiased selection method for resistance testing was applied, whereby either all patients meeting a defined VL threshold or a random selection were tested.

#### Types of Participants

Eligible studies investigated HIV type 1 (HIV-1)–infected participants aged >10 years [3] who received first-line ART with 2 NRTIs and 1 NNRTI for ≥6 months prior to switching to second-line ART, defined as ≥2 NRTIs with a ritonavir-boosted PI. Clinical, immunological, or virological criteria for switching to second-line ART were accepted, provided the criteria were clearly defined.

#### Analyses

The intention-to-treat (ITT) analysis described outcomes for all patients commencing second-line ART. Participants without virological data were categorized as lost to follow-up (no contact for ≥90 days since the last visit), died, transferred to another care provider, or missing data. The on-treatment analysis provided outcomes for participants who remained under follow-up with available VL results. For participants of observational studies who had commenced second-line ART but had not been in the study long enough to reach the virological analysis window, outcomes were imputed in proportion to the remaining participants in the cohort using a missing-at-random assumption. Data prior to imputation are presented in Supplementary Tables 2–3.

#### Virological Outcomes

The primary outcome was virological suppression, defined as plasma HIV-1 RNA <400 copies/mL after 48 and 96 weeks of second-line ART, with a 24-week window period to allow for variations across studies (eg, measurements taken between weeks 36 and 60 were accepted for the 48-week outcome). The 400 copies/mL threshold was chosen to reflect the most commonly used definition of virological suppression in studies from the region. Outcomes were further categorized as low-level viremia (400–1000 copies/mL) and virological failure as per WHO definition (>1000 copies/mL) [3].

A secondary analysis explored how detection of NRTI resistance prior to starting second-line ART influenced virological outcomes at week 48. We included studies with available data using an on-treatment analysis. The overall activity of the second-line regimen was scored as either full or partial using the Stanford Resistance algorithm (version 8.2) [9].

#### Resistance

The prevalence of major protease resistance mutations according to the Stanford Resistance algorithm (version 8.2) [9] after 48 and 96 weeks was calculated as a proportion of the population that underwent resistance testing at failure.

### Data Extraction

Following the literature search and removal of duplicate citations, 2 reviewers (A. J. S., M. J. S.) independently screened the abstracts of retrieved records to include all potentially relevant articles, and then independently reviewed the full text of the remaining articles. Disputes about inclusion of articles were resolved through discussion, with recourse to a third reviewer (A. M. G.). A. J. S. and M. J. S. independently extracted data from the studies.

### Quality Assessment

We conducted this study according to recommendations from the Preferred Reporting Items for Systematic Reviews and Meta-Analyses (PRISMA) guidelines [10]. The quality of included articles was assessed using a modified version of a quality appraisal tool (Supplementary Materials). The review was registered with PROSPERO (CRD42016048985).

### Statistical Analysis

Agreement between reviewers was assessed using Cohen κ statistic. Confidence intervals (CIs) were calculated using the Wilson method. Proportions were stabilized using the Freeman-Tukey arcsine square root transformation and a pooled proportion was calculated using the DerSimonian-Laird random effects model [11]. To assess the effect of preexisting NRTI resistance on virological outcomes, we calculated the odds ratio (OR) of pooled rates of virological suppression at 48 weeks among patients receiving fully active regimens compared to those on partially active regimens, using a DerSimonian-Laird random effects model. We reported the *I*^2^ statistic, where *I*^2^ is interpreted as the proportion of variability in the treatment estimate attributable to between-study heterogeneity rather than sampling error. We assessed potential publication bias by visual inspection of funnel plots and by Egger test [12].

To determine the effect on virological outcomes of study design (randomized vs observational), median CD4 cell count, year of study, and duration of first-line ART, we performed meta-regression analysis using a restricted maximum-likelihood estimator mixed effects model. Analyses were conducted in Stata version 14.2 software (StataCorp, College Station, Texas).

## RESULTS

### Data Selection and Quality Assessment: Virological Outcome Studies

**Figure 1.**
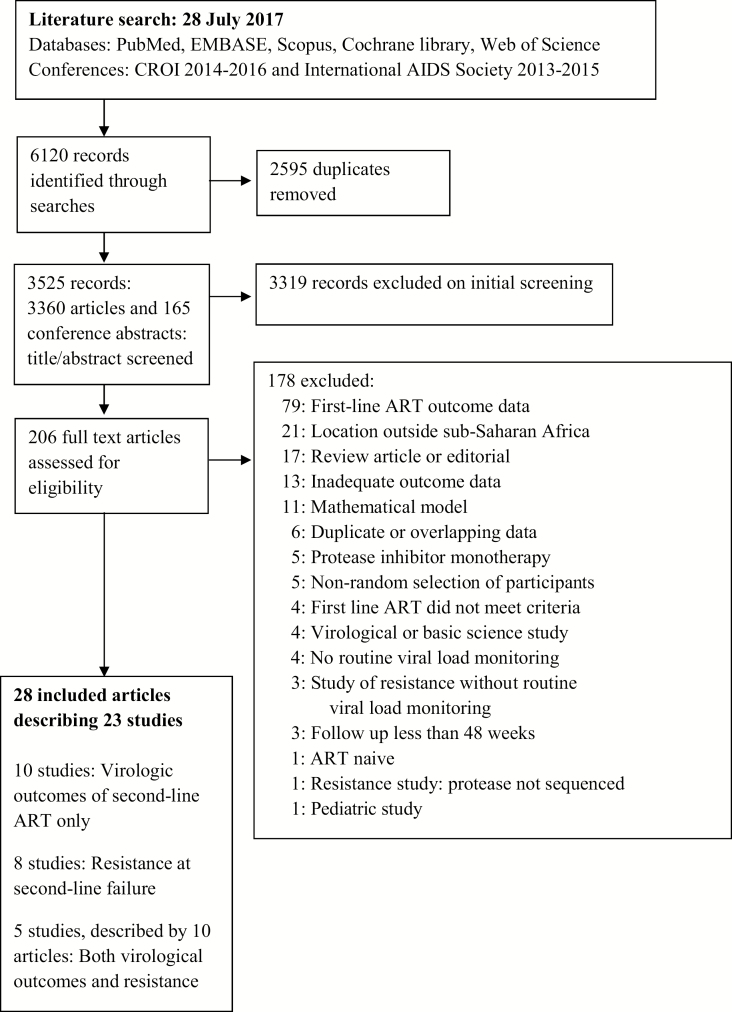
Flow diagram of search strategy. Abbreviations: ART, antiretroviral therapy; CROI, Conference on Retroviruses and Opportunistic Infections.

**Figure 2.**
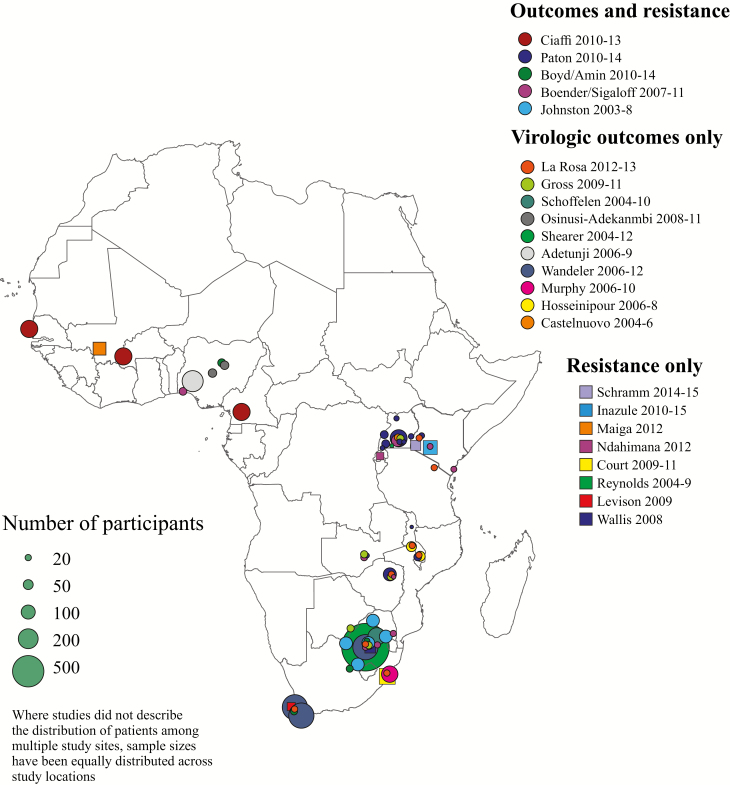
Map of included studies.

Following removal of duplicates, we screened 3525 abstracts and selected 206 full articles for review; the selection showed good agreement between reviewers (Cohen κ = 0.70 [95% CI, .63–.76]). Twenty articles describing 15 studies met the inclusion criteria (Figure 1), comprising 5 RCTs [6, 13–18], 5 prospective observational studies [19–25], and 5 retrospective observational studies [26–31]. Six studies were reported from multinational cohorts [13–17, 19, 23]. Data were available from 11 of 48 (23%) sub-Saharan African countries, with study locations in western, central, eastern, and southern Africa (Figure 2 and Table 1).

**Table 1.** Characteristics of Included Outcome Studies

Reference, First Author	Design	Year	Location	No.	Age, y, Median (IQR)	Gender, % Female	Duration of First-line ART, mo, Median (IQR)	Frequency of Viral Load Monitoring	Second-line ART				
									Switch Rate/1000 PY	Reason for Switch	PI	CD4 Count at Start, Cells/μL, Median (IQR)	Viral Load at Start, Log_10_ Copies/mL, Median (IQR)
La Rosa [17]	RCT	2012–13	Kenya, Malawi, South Africa, Tanzania, Zimbabwe	162	38 (33–43)	50	48 (26–72)	6 mo	NA	VF	LPV	182 (160)^a^	4.5 (0.9)^a^
Ciaffi [15]	RCT	2010–13	Cameroon, Senegal, Burkina Faso	451	38 (32–46)	72	49 (33–69)	3 mo	NA	VF	LPV 64%; DRV 34%	183 (87–290)	4.5 (4.0–51)
Paton [18]	RCT	2010–14	Uganda, Kenya, Malawi, Zimbabwe, Zambia	426	37 (31–43)	62	48 (34–65)	None	NA	VF	LPV	72 (29–143)	4.8 (4.4–5.2)
Boyd [14]; Amin [13]	RCT	2010–14	Nigeria, South Africa	100	38 (33–45)	65	29 (19–50)	3 mo	NA	VF	LPV	199 (64–284)	4.2 (3.5–4.9)
Gross [16]	RCT	2009–11	Botswana, South Africa, Uganda, Zambia, Zimbabwe	132	38 (34–45)	50	34 (20- 55)	3 mo	NA	VF	LPV	183 (94–271)	4.3 (3.8–4.9)
Osinusi-Adekanmbi [22]	POC	2008–11	Nigeria	73	35 (30–41)	67	24 (16–32)	6 mo	NA	VF	LPV	121	NA
Shearer [31]	ROC	2004–12	South Africa	1150	38 (33–44)	59	19 (13–31)	6 mo	NA	VF	LPV	203 (114–305)	4.2 (3.6–4.8)
Schoffelen [29]	ROC	2004–10	South Africa	156	35 (29–41)	72	19 (11–31)	6 mo	8	VF	LPV	187 (93–299)	4.0 (3.4–4.5)
Adetunji [26]	ROC	2006–09	Nigeria	225	34 (29–40)	65	16 (12–23)	6 mo	11	VF	LPV	139 (58–235)	4.6 (3.9–5.2)
Wandeler [24]	POC	2006–12	South Africa	971	38 (32–45)	56	27 (17–38)	6 mo	NA	C/I/VF	LPV	172 (95–267)	NA
Boender [19]; Sigaloff [23]	POC	2007–11	Kenya, Nigeria, Uganda, South Africa, Zambia, Zimbabwe	243	38 (34–45)	50	27 (15–44)	12 mo	32	C/I/VF	LPV	126 (66–205)	4.2 (3.2–5.0)
Murphy [28]	ROC	2006–10	South Africa	136	36 (31–43)	65	13 (7–20)	6 mo	10	VF	LPV	153 (89–232)	4.5 (3.8–4.9)
Johnston [27]	ROC	2003–08	South Africa	417	36 (31–44)	35	23 (15–34)	6 mo	6	VF	LPV	169 (97–235)	4.6 (4.1–5.1)
Hosseinipour [21]	POC	2006–08	Malawi	101	38 (32–46)	55	35 (25–49)	3 mo	8	VF	LPV	65 (22–173)	4.7 (4.1–5.2)
Castelnuovo [20]	POC	2004–06	Uganda	40	39 (36–43)	50	22 (19–23)	6 mo	47	VF	LPV	108 (43–205)	4.8 (4.0–5.4)

Abbreviations: ART, antiretroviral therapy; C, clinical failure; DRV, darunavir with ritonavir; I, immunological failure; IQR, interquartile range; LPV, lopinavir with ritonavir; NA, data not available; PI, protease inhibitor; PY, patient-years; POC, prospective observational cohort; RCT, randomized controlled trial; ROC, retrospective observational cohort; VF, virological failure.

^a^Mean (standard deviation).

Assessment of study quality is shown in Supplementary Table 4. The size of the initial first-line ART population, the rate of first-line ART failure, and the rate of switching to second-line ART were poorly described. The NRTIs used in first- and second-line regimens were inconsistently reported. The rate of adverse events and the contribution of tolerability to treatment discontinuation were not reported in most studies. In one study, criteria for starting second-line ART were at risk of performance bias as they included a requirement for regular attendance at clinic [20]. Sensitivity analysis excluding this trial from the ITT and on-treatment analyses did not significantly alter pooled estimates. There was no evidence of publication bias on inspection of funnel plots and by Egger test of asymmetry at 48 or 96 weeks (*P* = .16 and *P* =.19, respectively; Supplementary Figure 1).

### Outcomes of Second-line ART

**Figure 3.**
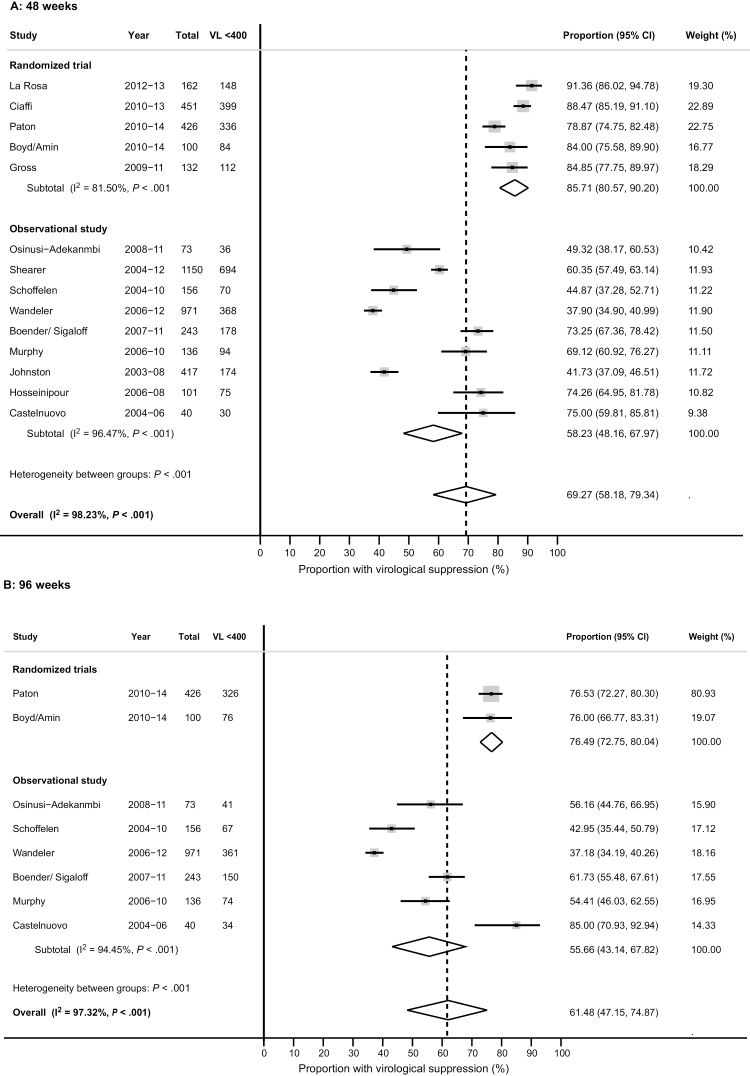
Forest plot of virological suppression at 48 weeks (*A*) and 96 weeks (*B*): intention–to-treat analysis, random effects model. Abbreviations: CI, confidence interval; VL <400, viral load <400 copies/mL.

**Figure 4.**
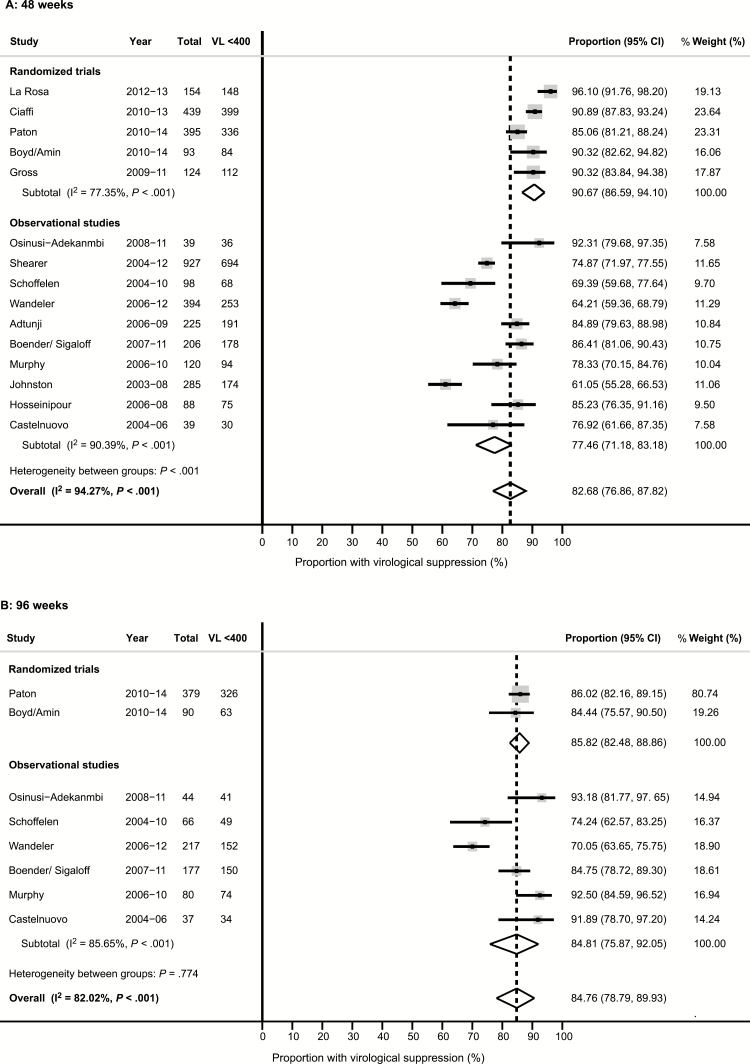
Forest plot of virological suppression at 48 weeks (*A*) and 96 weeks (*B*): on-treatment analysis, random effects model. Abbreviations: CI, confidence interval; VL <400, viral load <400 copies/mL.

The median duration of first-line ART prior to starting second-line ART varied from 13 to 49 months (Table 1). Estimates of the rate of switching from first-line to second-line ART were calculable for 8 studies and ranged from 6 to 47 per 1000 patient-years. All studies used twice-daily LPV/r; 1 RCT randomized one-third of participants to ritonavir-boosted darunavir (800 mg once daily) [15]. By ITT, virological suppression rates were 69.3% (95% CI, 58.2%–79.3%) among 4558 participants from 14 studies at week 48, and 61.5% (95% CI, 47.2%–74.9%) among 2145 participants from 8 studies at week 96 (Figure 3 and Supplementary Tables 2–3). In the on-treatment analysis, suppression rates were 82.7% (95% CI, 76.9%–87.8%) among 3626 participants from 15 studies at week 48, and 84.8% (95% CI, 78.8%–89.9%) among 1090 participants from 8 studies at week 96 (Figure 4 and Supplementary Table 5). The rate of virological failure according to the WHO definition (>1000 copies/mL) ranged between 2.5% and 26.6% of participants at 48 weeks and between 4.1% and 11.1% at 96 weeks, while low-level viremia occurred in 0–3.3% at 48 weeks and 0–5.0% at 96 weeks, respectively (Supplementary Tables 2–3).

Rates of virological suppression were significantly higher among participants of RCTs compared to observational cohorts at both week 48 (85.7% [95% CI, 80.6%–90.2%] vs 58.2% [95% CI, 48.2%–68.0%]; *P* < .001) and week 96 (76.5% [95% CI, 72.8%–80.4%] vs 55.7 [95% CI, 43.1%–67.8%]; *P* < .001). After exclusion of missing VL data, the difference between RCTs and observational cohorts persisted (*P* < .0001 and *P* = .001 at 48 and 96 weeks, respectively), and estimates of virological suppression rates did not significantly change (*P* = .39 and *P* = .58 at 48 and 96 weeks, respectively). By meta-regression analysis, neither median CD4 cell count, nor median duration of first-line ART at the time of starting second-line, nor the year of study recruitment were significantly associated with virological suppression, after adjustment for study design (*P* = .37, *P* = .83, and *P* = .95, respectively, at week 48; *P* = .91, *P* = .74, and *P* = .28, respectively, at week 96).

### Effect of Preexisting NRTI Resistance

**Figure 5.**
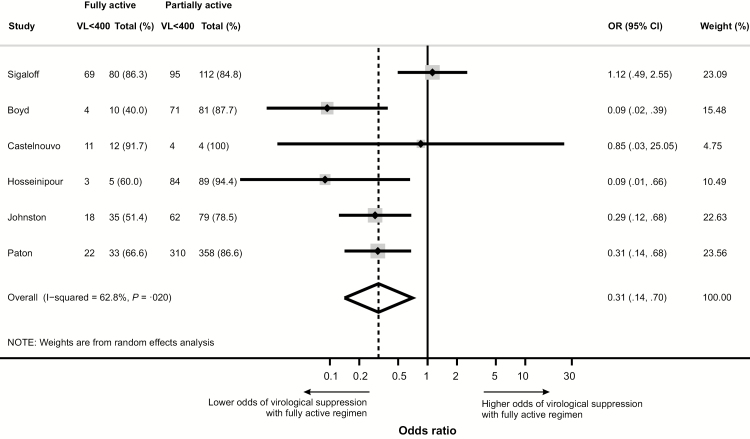
Forest plot: odds ratio for virological suppression at 48 weeks among participants with fully active compared to partially active second-line antiretroviral therapy (ART). Partially active ART is defined as low-level or greater resistance to any component of second-line ART (Stanford database version 8.2) [9]. Abbreviations: CI, confidence interval; OR, odds ratio; VL<400, viral load <400 copies/mL.

Resistance test results (by conventional sequencing) were available for 6 studies [6, 14, 18, 20, 21, 23, 30]. The likelihood of virological suppression at week 48 was lower (OR, 0.31 [95% CI, .14–.70]; *P* = .020) among participants lacking evidence of NRTI resistance and therefore predicted to be receiving fully active second-line ART, relative to those with NRTI resistance receiving partially active second-line ART (Figure 5). Preexisting NRTI resistance comprised predominantly the 3TC mutation M184V (67.0%–92.7% of participants) and thymidine analogue mutations (12.5%–74.3% of participants) (Supplementary Table 6).

### Protease Resistance at Failure of Second-line ART

**Figure 6.**
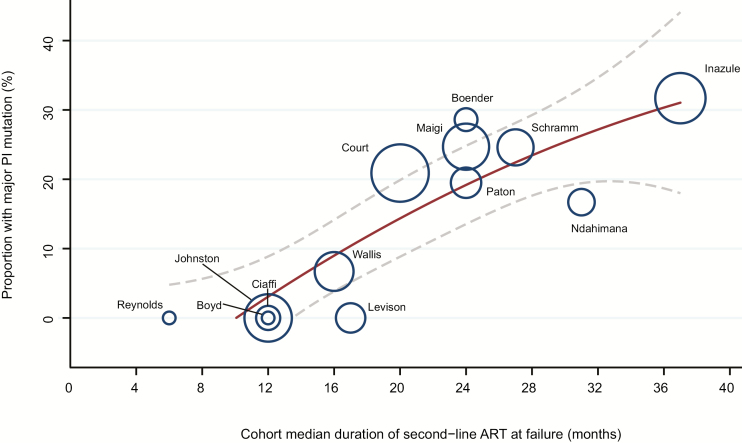
Proportion of participants with major protease mutations according to duration of second-line antiretroviral therapy at virological failure. Areas of circles are proportional to size of cohort failing second-line treatment. Solid line and dashed line are quadratic line of best fit and 95% confidence interval, respectively. Major protease resistance mutations according to the Stanford HIV resistance database version 8.2 [9]. Abbreviations: ART, antiretroviral therapy; PI, protease inhibitor.

Resistance test results (by conventional sequencing) were available from 649 participants from 13 studies, including 5 prospective [14, 15, 18, 23, 30] and 8 cross-sectional studies [32–39]. The threshold for resistance testing ranged from 400 to 5000 copies/mL. Duration of second-line ART at the time of sequencing ranged from 6 to 37 months. Major protease resistance mutations were present in a median of 17% (interquartile range, 0–25%; range, 0–66.7%) of patients who underwent resistance testing (Table 2). An association between the prevalence of protease resistance mutations and median duration of second-line ART was observed (0–11.8% at 6–12 months to 0–28.9% at 16–24 months, and 16.7%–66.7% at 27–37 months; *r*^2^ = 0.75, *P* < .001). (Figure 6).

**Table 2.** Protease Inhibitor Resistance at Failure of Second-line Antiretroviral Therapy

Reference	Study Design	Year	Location	Total Population, No.	Second-line ART Duration, mo, Median (IQR)	Viral Load Threshold for Sequencing, Copies/mL	Failure Population, No. (%)	Resistance Analysis Population, No. (%)	Protease Resistance, No. (% of At-Risk Population)^a^	Protease Resistance, No. (% of Those Sequenced)^b^	Major Protease Mutation (No.)
Prospective studies											
Paton [18]	RCT	2010–14	Uganda, Kenya, Malawi, Zimbabwe, Zambia	426	24	1000	46 (10.7)	41 (89.1)	8 (2.1)^c^	8 (19.5)^c^	M46I (8), I54V (7), L76V (3), V82AF (6)
Boyd [14]	RCT	2010–14	Nigeria, South Africa	100	12	500	8 (8.0)	8 (100)	0 (0)	0 (0)	…
Ciaffi [15]^d^	RCT	2010–13	Cameroon, Burkina Faso, Senegal	451	12	1000 × 2	29 (6.4)	5 (17.2)	0 (0)	0 (0)	…
Boender [25]	POC	2007–11	Kenya, Nigeria, South Africa, Uganda, Zambia, Zimbabwe	205	12	1000	21 (10.2)	17 (81.0)	2 (1.2)	2 (11.8)	M46I (2), I54V (2), L76V(1), V82A (2), L90M (1)
				177	24	1000	26 (14.7)	21 (80.8)	6 (4.2)	6 (28.6)	M46I (5), I54V (4), L76V(2), V82A (4), I84V (1)
				90	36	1000	8 (8.9)	3 (37.5)	2 (5.9)	2 (66.7)	M46I (2), I50V (1), I54V (1), V82A (2)
Johnston [30]	POC	2003–8	South Africa	417	12	400	112 (26.8)	15 (13.4)	0 (0)	0 (0)	…
Cross-sectional observational studies											
Schramm [39]	CS	2014–15	Kenya	355	27 (23–36)	500	65 (18.3)	65 (100)	16 (4.5)	16 (24.6)	NA
Inazule [38]	CS	2010–15	Kenya	NS	37 (23–55)	1000	126 (…)	123 (97.6)	39 (…)	39 (31.7)	M46I/L (30), I54V (27), V82ATFS (25)
Court [32]	CS	2009–13	South Africa	NS	20 (13–34)	1000	164 (…)	134 (81.7)	28 (…)	28 (20.9)	M46I (22), I47VA (2), I50V (1), I54VTALM (24), L76V (19), V82A (22), I84V (2), L90M (1)
Maiga [34]	CS	2012	Mali	913	24 (6–48)	500	106 (11.6)	93 (87.7)	23 (2.9)	23 (24.7)	M46I (15), I47V/A (6), I54V (12), L76V(11), V82A (8), I84V (10), L90M (3)
Ndahimana [37]	CS	2012	Rwanda	74	31 (18–46)	1000	35 (47.3)	30 (85.7)	5 (7.9)	5 (16.7)	L33F (2), M46I (4), I54V (5), L76V (2), V82A (4), I84V (2),
Levison [33]	CS	2009	South Africa	322	17 (18)^e^	1000 × 2	43 (13.3)	33 (76.7)	0 (0)	0 (0)	…
Reynolds [35]	CS	2004–9	Uganda	65	6 (6–14)	2000	8 (12.3)	6 (75.0)	0 (0)	0 (0)	…
Wallis [36]	CS	2008	South Africa	NS	16 (7–18)	5000 × 2	75 (…)	75 (100)	5 (…)	5 (6.7)	L33F (2), M46I (4), I54SV (2), L76V (2), V82A (1), I84V (2), L90M (1),

Abbreviations: ART, antiretroviral therapy; CS, cohort study; IQR, interquartile range; NA, genotype not available; NS, not specified; POC, prospective observational cohort; RCT, randomized controlled trial.

^a^As proportion of total at-risk population; adjusted for proportion who underwent sequencing. Major protease resistance mutations as defined by the Stanford HIV drug resistance database [9].

^b^As proportion of failure population; adjusted for proportion who underwent sequencing.

^c^Resistance refers to intermediate or high-level resistance to lopinavir only.

^d^All patients received lopinavir/ritonavir apart from participants in the Ciaffi et al study [15]; 33% were randomized to darunavir/ritonavir, and the remainder received lopinavir/ritonavir.

^e^Standard deviation.

## DISCUSSION

By 2030, the number of patients requiring second-line ART in sub-Saharan Africa is estimated to exceed 4 million [8]. Our pooled ITT estimates for virological suppression after 48 and 96 weeks of second-line ART were 69.3% and 61.5%, respectively, demonstrating reasonable efficacy of PI-based therapy with continued NRTI use in these treatment-experienced populations. Employing similar analytical methodologies, studies from India, China, and Cambodia reported virological suppression rates ranging from 70% to 85.7% over 48–96 weeks of second-line ART [40–42]. RCTs using LPV/r in high-income settings reported comparable virological suppression rates among treatment-experienced patients [43]. Rates of virological suppression with first-line ART in low- and middle-income countries were similar: 67.3% and 64.6% at weeks 48 and week 96, respectively [44]. Thus, first- and second-line ART regimens show overall comparable efficacy in sub-Saharan Africa, despite the widely held assumption that suboptimal adherence may drive first-line failure and continue to reduce responses after patients start second-line ART. Importantly, these rates fall considerably short of the 90% UNAIDS target for virological suppression. Use of a high-genetic-barrier regimen in first-line ART (eg, with dolutegravir) may be required to meet these targets [45]. Although options for first-line ART are expanding, evidence is presently limited for alternative second-line options [4].

One-third of participants did not achieve virological suppression. An important reason in the ITT analysis, and a source of significant heterogeneity between studies, was the proportion of missing VL data (excluding death or loss to follow-up), which varied from 0 to 30%, despite accepting a 24-week window. This finding implies substantial challenges in implementation of VL monitoring. Consistent with this observation, virological outcomes were significantly better and loss to follow-up was lower among RCT participants compared to those from observational studies, a finding that persisted after exclusion of missing VL data. In the Europe-Africa Research Network for Evaluation of Second-line Therapy (EARNEST) trial, therapy was delivered in a manner designed to replicate typical program settings with broadly generalizable entry criteria, predominantly nurse-led care and without real-time VL monitoring [18]. Outcomes were comparable to other trials with more restrictive entry criteria that used real-time VL monitoring. Enhanced attention to patient retention, improving staffing, and provision of a constant drug supply are important for ensuring improved treatment outcomes and are likely to account for the observed differences between RCTs and observational studies.

Emergence of drug resistance is common after failure of first-line ART and is typically characterized by mutations affecting both NNRTIs and NRTIs [46–51]. Interestingly, detection of NRTI resistance and, specifically, thymidine analogue mutations (TAMs) prior to starting second-line ART predicted significantly higher odds of virological suppression [5, 14, 20, 21, 23, 30]. An explanation is that patients who develop resistance at failure of first-line ART may have overall higher levels of adherence (and therefore greater drug selective pressure) than subjects who experience failure in the absence of resistance [5]. Importantly, the NRTIs commonly included in second-line regimens, such as zidovudine or TDF + 3TC, retain significant residual activity in the presence of TAMs and this is enhanced by continuation of 3TC [52, 53]. Data from the SECOND-LINE and EARNEST studies demonstrate that apparent paradoxical benefit of NRTI resistance persists at 96–144 weeks [5, 6].

Current reports of HIV epidemic control do not differentiate between first- and second-line ART provision, and rates of second-line failure are not included among metrics of epidemic control or ART program performance [54]. Yet, between 2% and 26% of recipients of second-line ART experienced virological failure by 48 weeks. The optimal public health management of second-line failure has not been adequately defined. In South Africa, 64% of patients experiencing viremia >400 copies/mL (median, 3.5 log_10_ copies/mL) while on second-line ART regained virological suppression 2–4 months after targeted adherence counseling [55]. This rate of resuppression is consistent with our finding that major protease resistance mutations were uncommon at virological failure, particularly in the first 18 months of second-line ART. Emphasis on adherence is therefore necessary for second-line recipients. This should be differentiated from first-line failure where rapid emergence of NNRTI resistance is likely to limit the impact of adherence support. Effective adherence interventions may include weekly SMS (ie, text messaging) reminders and targeted counseling [56]. In cohort studies from Cambodia [57], India [40], and Vietnam [58], higher rates (42%–68%) of major protease mutations were observed at failure of second-line ART. This higher rate may reflect differences in adherence, duration of failing regimens, or an effect of viral subtypes. In our analysis, rates of PI resistance were strongly associated with increasing duration of second-line ART, suggesting that duration of PI failure is an important determinant of the need for third-line ART. Optimizing the frequency of VL monitoring and the definition of virological failure for second-line ART and defining appropriate regimens for third-line ART represent clear research priorities.

There are a number of limitations in our analysis. First, there was substantial variation in both the duration of first-line ART at the time of switching to second-line ART and the rate of switching to second-line ART among each cohort, which was only reported in 8 studies. The lack of consistency may represent a source of reporting bias. The variation in rate of switching we observed across studies (range, 6–47 per 1000 person years) is consistent with other low- and middle-income settings [7]. In programs with routine VL monitoring, rates of switching are 3 times higher, suggesting potentially different outcomes in programs without monitoring [7]. Second, our analysis used aggregate rather than individual patient data and, therefore, it was not possible to analyze the contribution of individual risk factors to outcomes. Third, most studies applied a VL <400 copies/mL to denote suppression. Data from South Africa demonstrate a continuum of risk of virological failure even with the lowest level of viremia (50–199 copies/mL), indicating that low-level viremia should trigger adherence interventions and repeat VL measurement [59]. Fourth, zidovudine and stavudine, previously common components of ART regimens in sub-Saharan Africa, have now been replaced by TDF, and impact on NRTI resistance profiles and second-line ART efficacy is to be demonstrated [60].

In summary, reported rates of virological suppression among patients receiving second-line PI-based ART in sub-Saharan Africa are similar to those observed with first-line ART and comparable to the outcomes of similar regimens in Asian and Western settings. There is a significant gap in achieving the third part of the WHO 90-90-90 strategy for epidemic control. Reporting of second-line ART provision and rates of virological suppression among recipients is crucial to understanding of epidemic control and should be strongly encouraged. Given that more than one-third of patients did not achieve virological suppression, defining the optimal definition and management of second-line ART failure, both with and without PI resistance, in this setting is an urgent research priority.

## Supplementary Data

Supplementary materials are available at *Clinical Infectious Diseases* online. Consisting of data provided by the authors to benefit the reader, the posted materials are not copyedited and are the sole responsibility of the authors, so questions or comments should be addressed to the corresponding author.

Supplementary Data

## Notes

***Acknowledgments.*** The authors thank the Ndlovu Care Group, Elandsdoorn, Limpopo, South Africa, and all the participants and study teams involved in the included studies.

***Financial support.*** This work was supported by the Wellcome Trust (Clinical PhD Fellowships grant numbers 109130/Z/15/Z and 201251/Z/16/Z to A. J. S. and M. J. S.) and the National Institutes of Health (grant numbers AI069481, AI-27757, and AI068636 to A. C. C.).

***Potential conflicts of interest.*** M. A. B. has received grants from AbbVie, Gilead, and Merck, and personal fees from AbbVie, Gilead, Merck, and ViiV Healthcare. G. W. has received grants from Gilead, MSD, and Roche Diagnostics. A. C. C. has received grants from the National Institutes of Health, and personal fees from Merck & Co and the International Antiviral Society–USA. N. I. P. has received personal fees from AbbVie, Janssen, and Roche, and grants and nonfinancial support from GSK. A. M. G. has received consulting honoraria from AbbVie, BMS, Gilead, Janssen, Merck, and ViiV, and speaker’s fees from AbbVie, BMS, Gilead, Janssen, and ViiV. The University of Liverpool is the recipient of research grants from BMS, Gilead, Janssen, and ViiV of which A. M. G. is the principal investigator. A. M. G. is also employed as Expert Scientist by Roche Pharma Research and Early Development, Discovery and Translation Area (Immunology, Inflammation and Infectious Diseases). All other authors report no potential conflicts. All authors have submitted the ICMJE Form for Disclosure of Potential Conflicts of Interest. Conflicts that the editors consider relevant to the content of the manuscript have been disclosed.

## References

[1] Joint United Nations Programme on HIV/AIDS. Global AIDS update. Geneva, Switzerland: UNAIDS, 2016.

[2] Bor J, Herbst AJ, Newell ML, Bärnighausen T Increases in adult life expectancy in rural South Africa: valuing the scale-up of HIV treatment. Science 2013; 339:961–5. 23430655 10.1126/science.1230413 PMC3860268

[3] World Health Organization. Consolidated guidelines on the use of antiretroviral drugs for treating and preventing HIV infection. Geneva, Switzerland: WHO, 2016. 27466667

[4] Kanters S, Socias ME, Paton NI et al Comparative efficacy and safety of second-line antiretroviral therapy for treatment of HIV/AIDS: a systematic review and network meta-analysis. Lancet HIV 2017; 4:e433–41. 28784426 10.1016/S2352-3018(17)30109-1

[5] Boyd MA, Moore CL, Molina JM et al SECOND-LINE Study Group Baseline HIV-1 resistance, virological outcomes, and emergent resistance in the SECOND-LINE trial: an exploratory analysis. Lancet HIV 2015; 2:e42–51. 26424460 10.1016/S2352-3018(14)00061-7

[6] Paton NI, Kityo C, Thompson J et al Europe Africa Research Network for Evaluation of Second-line Therapy (EARNEST) Trial Team Nucleoside reverse-transcriptase inhibitor cross-resistance and outcomes from second-line antiretroviral therapy in the public health approach: an observational analysis within the randomised, open-label, EARNEST trial. Lancet HIV 2017; 4:e341–8. 28495562 10.1016/S2352-3018(17)30065-6 PMC5555436

[7] Haas AD, Keiser O, Balestre E et al IeDEA Southern Africa, East Africa, and West Africa Monitoring and switching of first-line antiretroviral therapy in adult treatment cohorts in sub-Saharan Africa: collaborative analysis. Lancet HIV 2015; 2:e271–8. 26423252 10.1016/S2352-3018(15)00087-9 PMC4500741

[8] Estill J, Ford N, Salazar-Vizcaya L et al The need for second-line antiretroviral therapy in adults in sub-Saharan Africa up to 2030: a mathematical modelling study. Lancet HIV 2016; 3:e132–9. 26939736 10.1016/S2352-3018(16)00016-3 PMC5688234

[9] Liu TF, Shafer RW Web resources for HIV type 1 genotypic-resistance test interpretation. Clin Infect Dis 2006; 42:1608–18. 16652319 10.1086/503914 PMC2547473

[10] Moher D, Liberati A, Tetzlaff J, Altman DG; PRISMA Group Preferred reporting items for systematic reviews and meta-analyses: the PRISMA statement. BMJ 2009; 339:b2535. 19622551 10.1136/bmj.b2535 PMC2714657

[11] Higgins JPT, Green S, eds. Cochrane Handbook for Systematic Reviews of Interventions Version 5.1.0 [Updated March 2011]. The Cochrane Collaboration, 2011. Available at: http://handbook.cochrane.org.

[12] Egger M, Davey Smith G, Schneider M, Minder C Bias in meta-analysis detected by a simple, graphical test. BMJ 1997; 315:629–34. 9310563 10.1136/bmj.315.7109.629 PMC2127453

[13] Amin J, Boyd MA, Kumarasamy N et al Raltegravir non-inferior to nucleoside based regimens in second-line therapy with lopinavir/ritonavir over 96 weeks: a randomised open label study for the treatment of HIV-1 infection. PLoS One 2015; 10:e0118228. 25723472 10.1371/journal.pone.0118228 PMC4344344

[14] Boyd M Ritonavir-boosted lopinavir plus nucleoside or nucleotide reverse transcriptase inhibitors versus ritonavir-boosted lopinavir plus raltegravir for treatment of HIV-1 infection in adults with virological failure of a standard first-line ART regimen (SECOND-LINE): a randomised, open-label, non-inferiority study. Lancet 2013; 381:2091–9. 23769235 10.1016/S0140-6736(13)61164-2

[15] Ciaffi L, Koulla-Shiro S, Sawadogo A et al 2LADY Study Group Efficacy and safety of three second-line antiretroviral regimens in HIV-infected patients in Africa. AIDS 2015; 29:1473–81. 26244387 10.1097/QAD.0000000000000709 PMC4502989

[16] Gross R, Zheng L, La Rosa A et al ACTG 5234 Team Partner-based adherence intervention for second-line antiretroviral therapy (ACTG A5234): a multinational randomised trial. Lancet HIV 2015; 2:e12–9. 10.1016/S2352-3018(14)00007-1 PMC4313760 26424232

[17] La Rosa AM, Harrison LJ, Taiwo B et al ACTG A5273 Study Group Raltegravir in second-line antiretroviral therapy in resource-limited settings (SELECT): a randomised, phase 3, non-inferiority study. Lancet HIV 2016; 3:e247–58. 27240787 10.1016/S2352-3018(16)30011-X PMC4914044

[18] Paton NI, Kityo C, Hoppe A et al EARNEST Trial Team Assessment of second-line antiretroviral regimens for HIV therapy in Africa. N Engl J Med 2014; 371:234–47. 25014688 10.1056/NEJMoa1311274

[19] Boender TS, Sigaloff KCE, Hamers RL et al Favorable long-term outcomes of 2nd-line ART despite drug-resistant HIV-1 in sub-Saharan Africa. Top Antivir Med 2014; 22.

[20] Castelnuovo B, John L, Lutwama F et al Three-year outcome data of second-line antiretroviral therapy in Ugandan adults: good virological response but high rate of toxicity. J Int Assoc Physicians AIDS Care (Chic) 2009; 8:52–9. 19095630 10.1177/1545109708328538

[21] Hosseinipour MC, Kumwenda JJ, Weigel R et al Second-line treatment in the Malawi antiretroviral programme: high early mortality, but good outcomes in survivors, despite extensive drug resistance at baseline. HIV Med 2010; 11:510–8. 20345885 10.1111/j.1468-1293.2010.00825.x PMC4833877

[22] Osinusi-Adekanmbi O, Stafford K, Ukpaka A et al Long-term outcome of second-line antiretroviral therapy in resource-limited settings. J Int Assoc Provid AIDS Care 2014; 13:366–71. 10.1177/2325957414527167 24668134

[23] Sigaloff KC, Hamers RL, Wallis CL et al PharmAccess African Studies to Evaluate Resistance (PASER) Second-line antiretroviral treatment successfully resuppresses drug-resistant HIV-1 after first-line failure: prospective cohort in sub-Saharan Africa. J Infect Dis 2012; 205:1739–44. 22448003 10.1093/infdis/jis261

[24] Wandeler G, Keiser O, Mulenga L et al IeDEA Southern Africa Collaboration Tenofovir in second-line ART in Zambia and South Africa: collaborative analysis of cohort studies. J Acquir Immune Defic Syndr 2012; 61:41–8. 22743595 10.1097/QAI.0b013e3182632540 PMC3432418

[25] Boender TS, Hamers RL, Ondoa P et al Protease inhibitor resistance in the first 3 years of second-line antiretroviral therapy for HIV-1 in sub-Saharan Africa. J Infect Dis 2016; 214:873–83. 27402780 10.1093/infdis/jiw219

[26] Adetunji AA, Achenbach C, Feinglass J et al Optimizing treatment switch for virologic failure during first-line antiretroviral therapy in resource-limited settings. J Int Assoc Provid AIDS Care 2013; 12:236–40. 23128403 10.1177/1545109712463733 PMC4604115

[27] Johnston V, Fielding K, Charalambous S et al Second-line antiretroviral therapy in a workplace and community-based treatment programme in South Africa: determinants of virological outcome. PLoS One 2012; 7:e36997. 22666338 10.1371/journal.pone.0036997 PMC3362581

[28] Murphy RA, Sunpath H, Castilla C et al Second-line antiretroviral therapy: long-term outcomes in South Africa. J Acquir Immune Defic Syndr 2012; 61:158–63. 22692090 10.1097/QAI.0b013e3182615ad1 PMC3767995

[29] Schoffelen AF, Wensing AM, Tempelman HA, Geelen SP, Hoepelman AI, Barth RE Sustained virological response on second-line antiretroviral therapy following virological failure in HIV-infected patients in rural South Africa. PLoS One 2013; 8:e58526. 23505529 10.1371/journal.pone.0058526 PMC3594302

[30] Johnston V, Cohen K, Wiesner L et al Viral suppression following switch to second-line antiretroviral therapy: associations with nucleoside reverse transcriptase inhibitor resistance and subtherapeutic drug concentrations prior to switch. J Infect Dis 2014; 209:711–20. 23943851 10.1093/infdis/jit411 PMC3923537

[31] Shearer K, Evans D, Moyo F et al Treatment outcomes of over 1000 patients on second-line, protease inhibitor-based antiretroviral therapy from four public-sector HIV treatment facilities across Johannesburg, South Africa. Trop Med Int Health 2017; 22:221–31. 27797443 10.1111/tmi.12804 PMC5288291

[32] Court R, Gordon M, Cohen K et al Random lopinavir concentrations predict resistance on lopinavir-based antiretroviral therapy. Int J Antimicrob Agents 2016; 48:158–62. 27345268 10.1016/j.ijantimicag.2016.04.030 PMC4979317

[33] Levison JH, Orrell C, Gallien S et al Virologic failure of protease inhibitor-based second-line antiretroviral therapy without resistance in a large HIV treatment program in South Africa. PLoS One 2012; 7:e32144. 22427821 10.1371/journal.pone.0032144 PMC3302781

[34] Maiga AI, Fofana DB, Cisse M et al Characterization of HIV-1 antiretroviral drug resistance after second-line treatment failure in Mali, a limited-resources setting. J Antimicrob Chemother 2012; 67:2943–8. 22888273 10.1093/jac/dks310 PMC3584968

[35] Reynolds SJ, Laeyendecker O, Nakigozi G et al Antiretroviral drug susceptibility among HIV-infected adults failing antiretroviral therapy in Rakai, Uganda. AIDS Res Hum Retroviruses 2012; 28:1739–44. 22443282 10.1089/aid.2011.0352 PMC3505045

[36] Wallis CL, Mellors JW, Venter WD, Sanne I, Stevens W Protease inhibitor resistance is uncommon in HIV-1 subtype C infected patients on failing second-line lopinavir/r-containing antiretroviral therapy in South Africa. AIDS Res Treat 2011; 2011:769627. 21490784 10.1155/2011/769627 PMC3066558

[37] Ndahimana Jd, Riedel DJ, Muhayimpundu R et al HIV drug resistance mutations among patients failing second-line antiretroviral therapy in Rwanda. Antivir Ther 2016; 21:253–9. 26562173 10.3851/IMP3005

[38] Inzaule SC, Hamers RL, Mukui I et al Emergence of untreatable, multidrug-resistant HIV-1 in patients failing second-line therapy in Kenya. AIDS 2017; 31:1495–8. 28398959 10.1097/QAD.0000000000001500

[39] Schramm B, Carnimeo V, Rakesh A et al Cross-sectional assessment of virological failure, drug resistance and third-line regimen requirements among patients receiving second-line ART in 3 large HIV programmes in Kenya, Malawi and Mozambique. J Int AIDS Soc 2016; 19.

[40] Chakravarty J, Sundar S, Chourasia A et al Outcome of patients on second line antiretroviral therapy under programmatic condition in India. BMC Infect Dis 2015; 15:517. 26572102 10.1186/s12879-015-1270-8 PMC4647630

[41] Ferradini L, Ouk V, Segeral O et al High efficacy of lopinavir/r-based second-line antiretroviral treatment after 24 months of follow up at ESTHER/Calmette Hospital in Phnom Penh, Cambodia. J Int AIDS Soc 2011; 14:14. 21439074 10.1186/1758-2652-14-14 PMC3072300

[42] Han Y, Li Y, Xie J et al Week 120 efficacy of tenofovir, lamivudine and lopinavir/r-based second-line antiretroviral therapy in treatment-experienced HIV patients. PLoS One 2015; 10:e0120705. 25821963 10.1371/journal.pone.0120705 PMC4379083

[43] Huang X, Xu Y, Yang Q et al Efficacy and biological safety of lopinavir/ritonavir based anti-retroviral therapy in HIV-1-infected patients: a meta-analysis of randomized controlled trials. Sci Rep 2015; 5:8528. 25704206 10.1038/srep08528 PMC4336931

[44] Boender TS, Sigaloff KC, McMahon JH et al Long-term virological outcomes of first-line antiretroviral therapy for HIV-1 in low- and middle-income countries: a systematic review and meta-analysis. Clin Infect Dis 2015; 61:1453–61. 26157050 10.1093/cid/civ556 PMC4599392

[45] Maartens G, Meintjes G Resistance matters in EARNEST. Lancet HIV 2017; 4:e323–4. 28495563 10.1016/S2352-3018(17)30087-5

[46] Boender TS, Kityo CM, Boerma RS et al Accumulation of HIV-1 drug resistance after continued virological failure on first-line ART in adults and children in sub-Saharan Africa. J Antimicrob Chemother 2016; 71:2918–27. 27342546 10.1093/jac/dkw218

[47] Guichet E, Aghokeng A, Serrano L et al Short communication: high viral load and multidrug resistance due to late switch to second-line regimens could be a major obstacle to reach the 90-90-90 UNAIDS objectives in Sub-Saharan Africa. AIDS Res Hum Retroviruses 2016; 32:1159–62. 27342228 10.1089/AID.2016.0010

[48] Sigaloff KC, Hamers RL, Wallis CL et al PharmAccess African Studies to Evaluate Resistance (PASER) Unnecessary antiretroviral treatment switches and accumulation of HIV resistance mutations; two arguments for viral load monitoring in Africa. J Acquir Immune Defic Syndr 2011; 58:23–31. 21694603 10.1097/QAI.0b013e318227fc34

[49] Barth RE, Aitken SC, Tempelman H et al Accumulation of drug resistance and loss of therapeutic options precede commonly used criteria for treatment failure in HIV-1 subtype-C-infected patients. Antivir Ther 2012; 17:377–86. 22297391 10.3851/IMP2010

[50] Hamers RL, Sigaloff KC, Wensing AM et al PharmAccess African Studies to Evaluate Resistance (PASER) Patterns of HIV-1 drug resistance after first-line antiretroviral therapy (ART) failure in 6 sub-Saharan African countries: implications for second-line ART strategies. Clin Infect Dis 2012; 54:1660–9. 22474222 10.1093/cid/cis254

[51] Lam EP, Moore CL, Gotuzzo E et al Antiretroviral resistance after first-line antiretroviral therapy failure in diverse HIV-1 subtypes in the SECOND-LINE Study. AIDS Res Hum Retroviruses 2016; 32:841–50. 27346600 10.1089/AID.2015.0331

[52] Deeks SG, Hoh R, Neilands TB et al Interruption of treatment with individual therapeutic drug classes in adults with multidrug-resistant HIV-1 infection. J Infect Dis 2005; 192:1537–44. 16206068 10.1086/496892

[53] Campbell TB, Shulman NS, Johnson SC et al Antiviral activity of lamivudine in salvage therapy for multidrug-resistant HIV-1 infection. Clin Infect Dis 2005; 41:236–42. 15983922 10.1086/430709

[54] Assefa Y, Gilks CF Second-line antiretroviral therapy: so much to be done. Lancet HIV 2017; 4:e424–5. 28784425 10.1016/S2352-3018(17)30112-1

[55] Fox MP, Berhanu R, Steegen K et al Intensive adherence counselling for HIV-infected individuals failing second-line antiretroviral therapy in Johannesburg, South Africa. Trop Med Int Health 2016; 21:1131–7. 27383454 10.1111/tmi.12741

[56] Mills EJ, Lester R, Thorlund K et al Interventions to promote adherence to antiretroviral therapy in Africa: a network meta-analysis. Lancet HIV 2014; 1:e104–11. 26424119 10.1016/S2352-3018(14)00003-4 PMC5096455

[57] Nerrienet E, Nouhin J, Ngin S et al HIV-1 protease inhibitors resistance profiles in patients with virological failure on LPV/r-based 2nd line regimen in Cambodia. J AIDS Clin Res 2012; S5:003.

[58] Thao VP, Quang VM, Day JN et al High prevalence of PI resistance in patients failing second-line ART in Vietnam. J Antimicrob Chemother 2016; 71:762–74. 26661398 10.1093/jac/dkv385 PMC4743698

[59] Hermans LE, Moorhouse M, Carmona S et al Effect of HIV-1 low-level viraemia during antiretroviral therapy on treatment outcomes in WHO-guided South African treatment programmes: a multicentre cohort study [manuscript published online ahead of print 17 November 2017]. Lancet Infect Dis 2017. doi:10.1016/S1473-3099(17)30681-3. 10.1016/S1473-3099(17)30681-3 29158101

[60] Gregson J, Kaleebu P, Marconi VC et al Occult HIV-1 drug resistance to thymidine analogues following failure of first-line tenofovir combined with a cytosine analogue and nevirapine or efavirenz in sub Saharan Africa: a retrospective multi-centre cohort study. Lancet Infect Dis 2017; 17:296–304. 27914856 10.1016/S1473-3099(16)30469-8 PMC5421555

