# Supplementary material for: Effectiveness of Protease Inhibitor/Nucleos(t)ide Reverse Transcriptase Inhibitor–Based Second-line Antiretroviral Therapy for the Treatment of Human Immunodeficiency Virus Type 1 Infection in Sub-Saharan Africa: A Systematic Review and Meta-analysis
Source: Clin Infect Dis. 2017 Dec 20;66(12):1846–57. doi: 10.1093/cid/cix1108 (PMC5982734; doi:10.1093/cid/cix1108)
Supplement: Supplementary Data [file cix1108_suppl_supplementary_data.pdf]

SUPPLEMENTARY DATA

Supplementary Table 1: Example search strategy

**Search**	**Query**
#4	Search (#1 AND #2 AND #3)
#3	Search "HIV Infections"[MeSH] OR HIV[MeSH] OR HIV[tiab] OR hiv-1*[tiab] OR hiv1[tiab] OR HIV infect*[tiab] OR human immunodeficiency virus[tiab] OR human immunedeficiency virus[tiab] OR human immuno-deficiency virus[tiab] OR human immunedeficiency virus[tiab] OR ((human immun*) AND (deficiency virus[tiab])) OR acquired immunodeficiency syndrome[tiab] OR acquired immunedeficiency syndrome[tiab] OR acquired immuno-deficiency syndrome[tiab] OR acquired immune-deficiency syndrome[tiab] OR ((acquired immun*) AND (deficiency syndrome[tiab]))
#2	Search "second line"[tiab] OR "second-line"[tiab] or “second line treatment”[tiab] OR “second-line treatment”[tiab] or “second-line antiretroviral therapy”[tiab] OR “second line antiretroviral therapy”[tiab] OR "protease inhibitor*"[tiab] OR “HIV Protease Inhibitor”[MeSH] OR "integrase inhibitor"[tiab] OR “HIV Integrase Inhibitors”[MeSH] OR lopinavir[tiab] OR lopinavir/ritonavir[tiab] OR atazanavir[tiab] OR darunavir[tiab] OR ritonavir[tiab] OR raltegravir[tiab] OR dolutegravir[tiab] OR indinavir[tiab] OR nelfinavir[tiab] OR fosamprenavir[tiab] OR etravirine[tiab] OR “treatment failure”[MeSH] OR stribild[tiab] OR eviplera[tiab] OR complera[tiab] OR maraviroc[tiab]
#1	Search Africa[MeSH] OR africa[tiab] OR "sub Saharan africa"[tiab] OR "sub-Saharan Africa"[tiab] OR Angola[tiab] OR Benin[tiab] OR Botswana[tiab] OR "Burkina Faso"[tiab] OR Burundi[tiab] OR Cameroon[tiab] OR "Cape Verde"[tiab] OR "Central African Republic"[tiab] OR Chad[tiab] OR Comoros[tiab] OR "Republic of the Congo"[tiab] OR "Democratic Republic of the Congo"[tiab] OR "Cote d'Ivoire"[tiab] OR Djibouti[tiab] OR "Equatorial Guinea"[tiab] OR Eritrea[tiab] OR Ethiopia[tiab] OR Gabon[tiab] OR "The Gambia"[tiab] OR Ghana[tiab] OR Guinea[tiab] OR "Guinea-Bissau"[tiab] OR Kenya[tiab] OR Lesotho[tiab] OR Liberia[tiab] OR Madagascar[tiab] OR Malawi[tiab] OR Mali[tiab] OR Mauritania[tiab] OR Mauritius[tiab] OR Mozambique[tiab] OR Namibia[tiab] OR Niger[tiab] OR Nigeria[tiab] OR Rwanda[tiab] OR "Sao Tome and Principe"[tiab] OR Senegal[tiab] OR Seychelles[tiab] OR "Sierra Leone"[tiab] OR "South Africa"[tiab] OR "South Sudan"[tiab] OR Sudan[tiab] OR Swaziland[tiab] OR Tanzania[tiab] OR Togo[tiab] OR Uganda[tiab] OR Zambia[tiab] OR Zimbabwe[tiab]

[tiab] = title and abstract [MeSH]= medline search heading

Supplementary Table 2: Outcomes of second-line ART at 48 weeks, ITT analysis

Study/Reference	Number of participants	Virological outcome, n (%)			No virological data, n (%)					Alive, receiving care, n (%)
		Suppression	LLV	VF	Dead	TO	LTFU	Missing data	IFU	
**Randomised controlled trials**										
La Rosa[1]	162	148 (91.4)	2 (1.2)	4 (2.5)	2 (1.2)	0 (0)	3 (1.9)	3 (1.9)	-	157/162 (96.9)
Ciaffi[2]	451	399 (88.5)	11 (2.4)	29 (6.4)	6 (1.3)	0 (0)	4 (0.9)	2 (0.4)	-	441/451 (97.8)
Paton[3]	426	336 (78.9)	14 (3.3)	45 (10.6)	21 (4.9)	0 (0)	0 (0)	10 (2.3)	-	405/426 (95.1)
Boyd[4]/Amin[5]	100	84 (84.0)	1 (1.0)	8 (8.0)	4 (4.0)	1 (1.0)	1 (1.0)	1 (1.0)	-	94/100 (94.0)
Gross[6]	132	112 (84.8)	3 (2.2)	9 (6.8)	4 (3.0)	1 (0.8)	0 (0)	3 (2.3)	-	127/132 (96.2)
**Prospective/ retrospective observational cohort**										
Osinusi-Adekanmbi[7]*	73	36 (49.3)	-	3 (4.1)	3 (4.1)	5 (6.8)	4 (5.5)	22 (30.1)	0 (0)	61/73 (83.6)
Shearer[8]*	1150	694 (60.3)	-	233(20.2)	22 (1.9)	0 (0)	123 (10.9)	223 (19.3)	0 (0)	1005/1150 (87.4)
Schoffelen[9]	156	68 (43.6)	2 (1.3)	28 (17.9)	4 (2.6)	4 (2.6)	17 (10.9)	30 (21.2)	3 (1.9)	128/153 (83.7)
Wandeler[10]	971	253 (26.1)	21 (2.2)	120 (12.4)	22 (2.3)	0 (0)	49 (5.0)	295 (30.3)	211 (21.7)	689/760 (90.7)
Boender[11]/Sigaloff[12]	243	178 (73.3)	8 (3.3)	20 (8.2)	10 (4.1)	5 (2.1)	15 (6.1)	7 (2.8)	0 (0)	213/243 (87.7)
Murphy[13]	136	94 (69.1)	0 (0)	26 (19.1)	0 (0)	6 (4.4)	4 (2.9)	6 (4.4)	0 (0)	126/136 (92.6)
Johnston[14, 15]*	417	174 (41.7)	-	111 (26.6)	19 (4.6)	23 (5.6)	29 (7.0)	61 (14.6)	-	346/417 (83.0)
Hosseinipour[16]	101	75 (74.2)	-	13 (12.8)	10 (9.9)	0 (0)	3 (3.0)	0 (0)	0 (0)	88/101 (87.1)
Castelnuovo[17]	40	30 (75.0)	0 (0)	9 (22.5)	1 (2.5)	0 (0)	0 (0)	0 (0)	0 (0)	39/40 (97.5)

ITT= Intention-to-treat; LLV= low level viraemia (VL 400-999 copies/ml); VF= Virological failure (>1000 copies/ml); TO= transferred out; LTFU= Lost to follow up; IFU= Insufficient follow up the proportion of patients in prospective observational cohorts who had not accrued sufficient follow-up at the time of analysis.

*In the indicated cohorts, viral load data was dichotomised at the 400 copies/ml cut-off; data was not available for the proportion with LLV; VF is defined as >400 copies/ml

Supplementary Table 3: Outcomes of second-line ART at 96 weeks, ITT analysis

Study/ Reference	Number of participants	Virological outcome, n (%)			No virological data, n (%)					Alive, receiving care
		Suppression	LLV	VF	Dead	TO	LTFU	Missing data	IFU	
**Randomised controlled trials**										
Paton[3]	426	326 (76.5)	7 (1.6)	46 (10.8)	30 (7.0)	0 (0)	4 (0.9)	13 (3.1)	-	392/426 (92.0)
Amin[5]	100	76 (76.0)	5 (5.0)	9 (9.0)	5 (5.0)	1 (1.0)	2 (2.0)	2 (2.0)	-	92/100 (92.0)
**Prospective/ retrospective observational cohort**										
Osinusi-Adekanmbi[7]	73	41 (56.2)	0 (0)	3 (4.1)	5 (6.8)	6 (8.2)	6 (8.2)	12 (16.4)	0 (0)	56/73 (76.7)
Schoffelen[9]	156	49 (31.4)	2 (1.3)	15 (9.6)	8 (5.1)	9 (5.8)	24 (15.4)	10 (6.4)	39 (25.0)	76/117 (65.0)
Wandeler[10]	971	152 (15.7)	7 (0.7)	58 (6.0)	39 (4.0)	0 (0)	67 (6.9)	204 (21.0)	444 (45.7)	421/527 (80.0)
Boender[11]/Sigaloff[12]	243	150 (61.7)	0 (0)	27 (11.1)	14 (5.8)	13 (5.3)	24 (9.9)	15 (6.2)	0 (0)	192/243 (79.0)
Murphy[13]	136	74 (54.4)	0 (0)	6 (4.4)	1 (0.7)	19 (14.0)	17 (12.5)	19 (14.0)	0 (0)	99/136 (72.8)
Castelnuovo[17]	40	34 (85.0)	0 (0)	3 (7.5)	1 (2.5)	0 (0)	2 (5.0)	0 (0)	0 (0)	37/40 (92.5)

ITT= Intention-to-treat; LLV= low level viraemia (VL 400-999 copies/ml); VF= Virological failure (>1000 copies/ml); TO= transferred out; LTFU= Lost to follow up; IFU= Insufficient follow up the proportion of patients in prospective observational cohorts who had not accrued sufficient follow-up at the time of analysis.

Supplementary Table 4: Quality assessment of included studies

Study/ Ref	Participant characteristics adequately described?	Eligibility criteria explicit & appropriate?	Participant recruitment/ selection adequately described?	Intervention clearly described?	Co-interventions clearly reported?	Outcome measure defined?	Outcomes measured before & after intervention?	Length of follow up reported?	Loss to follow up reported?	Adverse events reported?	Competing interests/ support reported?
La Rosa[1]	Y	Y	Y	Y	Y	Y	Y	Y	Y	Y	Y
Ciaffi[2]	Y	Y	Y	Y	Y	Y	Y	Y	Y	Y	Y
Paton[3]	Y	Y	Y	Y	Y	Y	Y	Y	Y	Y	Y
Boyd[4]/ Amin[5]	Y	Y	Y	Y	N	Y	Y	Y	Y	Y	Y
Gross[6]	Y	Y	Y	Y	Y	Y	Y	Y	Y	Y	Y
Osinusi-Adekanmbi[7]	Partially reported	Y	Y	Y	N	Y	Y	Y	Y	N	Y
Shearer[10]	Partially reported	Y	Y	Y	Y	Y	Y	Y	Y	N	Y
Schoffelen[9]	Y	Y	Y	Y	N	Y	Y	Y	Y	N	Y
Adetunji[18]a	Partially reported	Y	Y	N	N	Y	Y	Y	N[a]	N	Y
Wandeler[10]	Partially reported	Y	Y	N	N	Y	Y	Y	Y	N	Y
Boender[11]/ Sigaloff[12]	Y	Y	Y	N	N	Y	Y	Y	Y	N	Y
Murphy[13]	Partially reported	Y	Y	N	N	Y	Y	Y	Y	N	Y
Johnston[14, 15]	Y	Y	Y	N	Y	Y	Y	Y	Y	Y	Partially reported
Hosseinipour[16]	Y	Y	Y	Y	N	Y	Y	Y	Y	Y	Partially reported
Castelnuovo[17]	Partially reported	N	N	Y	N	Y	Y	Y	Y	Y	Y

Abbreviations: Y yes; N no.

[a]Adetunji et al included only participants with a known virological outcome at 12months; therefore study data was included only in on-treatment analyses

Supplementary Table 5: On-treatment outcomes of second-line ART at 48 and 96 weeks

Study/ Reference	Virological outcomes at 48 weeks, n (%)				Virological outcomes at 96 weeks, n (%)			
	Total	Suppression	LLV	VF	Total	Suppression	LLV	VF
**Randomised controlled trials**								
La Rosa[1]	154	148 (96.1)	2 (1.3)	4 (2.5)				
Ciaffi[2]	439	399 (90.9)	11 (2.5)	29 (6.6)				
Paton[3]	395	336 (85.1)	14 (3.5)	45 (11.4)	379	326 (86.0)	7 (1.8)	46 (12.1)
Boyd[4]/ Amin[5]	93	84 (90.3)	1 (1.1)	8 (8.6)	90	76 (84.4)	5 (5.6)	9 (10.0)
Gross[6]	124	112 (90.3)	3 (2.4)	9 (7.3)				
**Prospective/ retrospective observational cohort**								
Osinusi-Adekanmbi[7]*	39	36 (92.3)	-	3 (7.7)	44	41 (93.2)	-	3 (6.8)
Shearer[8]*	927	694 (74.9)	-	233 (25.1)				
Schoffelen[9]	98	68 (69.4)	2 (2.0)	28 (28.6)	66	49 (74.2)	2 (3.0)	15 (22.7)
Wandeler[10]	394	253 (64.2)	21 (5.3)	120 (30.5)	217	152 (70.0)	7 (3.2)	58 (26.7)
Adetunji[18][a]	225	191 (84.9)	0 (0)	34 (15.1)				
Boender[11]/ Sigaloff[12]	206	178 (86.4)	8 (3.9)	20 (9.7)	177	150 (84.7)	0 (0)	27 (15.3)
Murphy[13]	120	94 (78.3)	0 (0)	26 (21.7)	80	74 (92.5)	0 (0)	6 (7.5)
Johnston[14, 15]*	285	174 (61.1)	-	111 (38.9)				
Hosseinipour[16]*	88	75 (85.2)	-	13 (14.8)				
Castelnuovo[17]	39	30 (76.9)	0 (0)	9 (23.1)	37	34 (91.9)	0 (0)	3 (8.1)

LLV low level viraemia (VL 400-1000 copies/ml); VF= Virological failure;
*In the indicated cohorts, viral load data was dichotomised at 400 copies/ml; data were not available for the proportion with LLV; VF is defined as >400
a In Adetjunji *et al*, only patients with a known viral load at 12 months were included; the study is therefore only included in the on-treatment analysis.

Supplementary Table 6: List of baseline NRTI mutations in included studies

Study	Duration 1st line ART (months), median (IQR)	Sequences available	GenBank accession #[a]	M184VI, n(%)	TAMs, n(%)
Paton[19]	48 (34,65)	787[a]	KY061369 KY062155	730 (92.7)	585 (74.3)
Sigaloff[12]	27 (15, 44)	183	JN132214-JN132396	150 (82.0)	92 (50.3)
Boyd[20]	40 (22, 65)	91	-	77 (84.6)	25 (27.47)
Castelnouvo[17]	22 (19, 29)	16	-	13 (81.3)	2 (12.5)
Hosseinipour[16, 21]	37 (27, 50)	96	-	77 (80.2)	54 (56.3)
Johnston[14]	18 (10, 27)	115	KC921018-KC921144	77 (67.0)	39 (33.9)

[a] Sequences available at: https://www.ncbi.nlm.nih.gov/nucleotide/ Abbreviations: NRTI nucleoside reverse transcriptase inhibitor, IQR Interquartile range, TAM Thymidine analogue mutations, according to the Stanford HIV database (8.2)

Supplementary Figure 1: Funnel plots of studies included in intention to treat analysis of virologic suppression at 48 weeks (A) and 96 weeks (B)

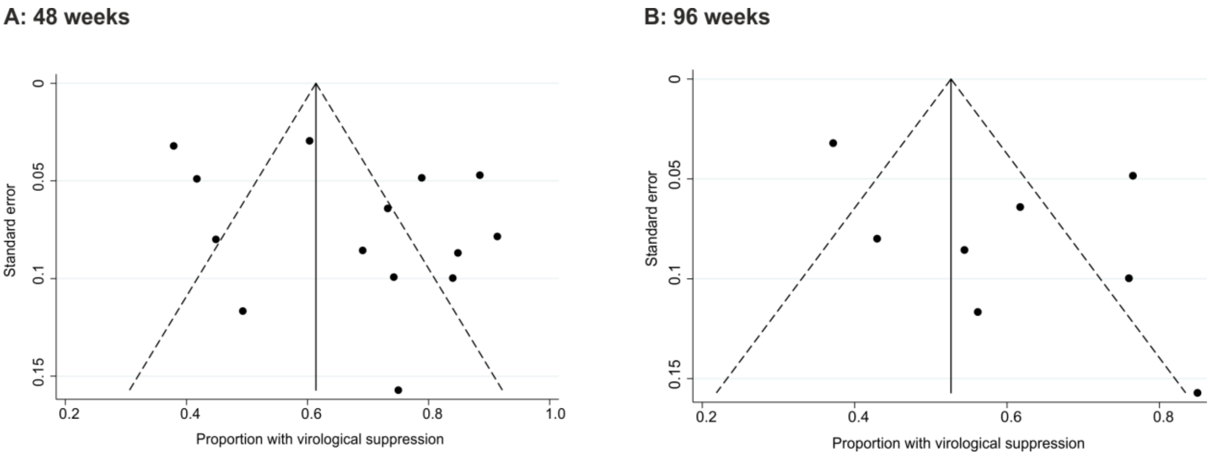

Supplementary Material: Quality assessment checklist

Adapted from: Development of a quality appraisal tool for case series using a modified Delphi technique (Institute of Health Economics) 2012[22]

Study population

1. Are the characteristics of the participants included in the study described?
Yes: The authors should report the total number, age, and gender distribution of the participants commencing second line ART, the duration and type of prior ART use, CD4 count and viral load at start of 2nd line therapy, if these criteria were used to define treatment failure.
Partially reported: The criteria above are incompletely reported
No: None of the relevant characteristics of the participants is reported.

2. Are the eligibility criteria (inclusion and exclusion criteria) to entry the study explicit and appropriate?
Yes: The eligibility criteria are clearly stated and replicable. A statement on age of eligibility, type of prior ART use accepted, definition of 1st line treatment failure are required.
No: The eligibility criteria are not clearly stated or are inappropriate.

3. Was the method of recruitment adequately described and appropriate?
Yes: There is a clear statement of the method of selection of participants. If viral load testing occurred, all patients received viral load testing or a clear description of the reason for missing data was given. For resistance studies, all participants who were virologically failing underwent resistance genotyping or they were a random unbiased selection of those failing and consistent virological thresholds for sequencing were applied.
Unclear: The method used to recruit participants is not clearly stated or no information is provided about the method used to recruit participants in the study. The method of selection for viral load testing was not described. Selection for resistance genotyping was vague or uncertain.
No: The participants were recruited based on other criteria, such as access to intervention or availability of resources, that could lead to a selection bias. Viral load or genotyping was not random and depended on availability or access to resources.

Intervention and co-intervention

4. Was the intervention clearly described in the study?
Yes: There is a detailed description about the characteristics of the intervention. The type and where non-standard, the dose and frequency of 2nd line ART should be described including details on choice of NRTIs.
No: The information provided is unclear, or important parameters of the intervention are missing from the presentation.

5. Were additional interventions (co-interventions) adequately reported in the study?
Yes: Co-interventions such as co-trimoxazole preventative therapy and any additional adherence support or increased clinic monitoring provided alongside 2nd line therapy should be described or if routine care was offered this should be described.
No: The use of co-interventions is not adequately described.

Outcome measures

6. Are the outcome measures clearly defined in the introduction or methods section?
Yes: All relevant (primary and secondary) outcomes that match the objective(s) of the study are described in the introduction or methods section. If virological/clinical/immunological failure, this must be defined (threshold, type and timing of tests). If loss to follow up is provided as an outcome this should be defined.
Partially reported: Some of the relevant outcomes are briefly reported in the introduction or methods section.
No: The outcomes are reported for the first time in the results or conclusion section of the study. The relevant outcomes are briefly mentioned without any details in the results, discussion, or conclusion section(s)**.** The outcomes reported are not relevant to study objective(s).

7. Were outcomes measured before and after intervention?
Yes: The relevant outcomes (viral load) are measured before and after applying the intervention.
Unclear: It is unclear when the outcomes were measured.
No: The outcomes are measured only after applying the intervention.

Results and conclusions

8. Was the length of follow-up reported?
Yes: The length of follow-up is clearly reported.
Unclear: The duration of follow-up is not clearly reported.
No: The length of follow-up is not reported, or the duration of the study is unclear.

9. Was the loss to follow-up reported?
Yes: The number or proportion of patients lost to follow-up is reported.
Unclear: It is not clear from the information provided how many participants were lost to follow-up or it is an inconsistence of reporting lost to follow-up (e.g. discrepancies between information from tables and text).
No: The number or proportion of patients lost to follow-up is not reported.

10. Are adverse events reported?
Yes: The undesirable or unwanted consequences of the intervention during the study period or within a prespecified time period are reported. Absence of any adverse event(s) is acknowledged in the study.
Partially reported: It is deducible that only some but not all potential adverse events are reported.
No: There is no statement about the presence or absence of adverse events.

Competing interest and source of support

11. Are both competing interest and source of support for the study reported?
Yes: Both competing interest and source of support (financial or other) received for the study are reported, or the absence of any competing interest and source of support is acknowledged.
Partially reported: Only one of these elements is reported.
No: Either there is no information available about competing interests and sources of support, or only one of these elements is reported.

Supplementary Material: List of major protease resistance mutations

The following mutations were designated as major protease resistance mutations in this study, as defined by the Stanford HIV database[23]:

D30N, V32I, L33F, M46IL, I47VA, G48VM, I50LV, I54VTALM, L76V, V82ATFS, I84V, N88S and L90M

REFERENCES

1. La Rosa AM, Harrison LJ, Taiwo B, et al. Raltegravir in second-line antiretroviral therapy in resource-limited settings (SELECT): a randomised, phase 3, non-inferiority study. Lancet HIV **2016**; 3(6): e247-58.
2. Ciaffi L, Koulla-Shiro S, Sawadogo A, et al. Efficacy and safety of three second-line antiretroviral regimens in HIV-infected patients in Africa. Aids **2015**; 29(12): 1473-81.
3. Paton NI, Kityo C, Hoppe A, et al. Assessment of second-line antiretroviral regimens for HIV therapy in Africa. N Engl J Med **2014**; 371(3): 234-47.
4. Boyd M. Ritonavir-boosted lopinavir plus nucleoside or nucleotide reverse transcriptase inhibitors versus ritonavir-boosted lopinavir plus raltegravir for treatment of HIV-1 infection in adults with virological failure of a standard first-line ART regimen (SECOND-LINE): A randomised, open-label, non-inferiority study. The Lancet **2013**; 381(9883): 2091-9.
5. Amin J, Boyd MA, Kumarasamy N, et al. Raltegravir non-inferior to nucleoside based regimens in second-line therapy with lopinavir/ritonavir over 96 weeks: a randomised open label study for the treatment of HIV-1 infection. PLoS One **2015**; 10(2): e0118228.
6. Gross R, Zheng L, Rosa AL, et al. Partner-based adherence intervention for second-line antiretroviral therapy (ACTG A5234): A multinational randomised trial. Lancet HIV Vol. 2, **2015**:e12-e9.
7. Osinusi-Adekanmbi O, Stafford K, Ukpaka A, et al. Long-term outcome of second-line antiretroviral therapy in resource-limited settings. J Int Assoc Provid AIDS Care **2014**; 13(4): 366-71.
8. Shearer K, Evans D, Moyo F, et al. Treatment outcomes of over 1000 patients on second-line, protease inhibitor-based antiretroviral therapy from four public-sector HIV treatment facilities across Johannesburg, South Africa. Trop Med Int Health **2017**; 22(2): 221-31.
9. Schoffelen AF, Wensing AM, Tempelman HA, Geelen SP, Hoepelman AI, Barth RE. Sustained virological response on second-line antiretroviral therapy following virological failure in HIV-infected patients in rural South Africa. PLoS One **2013**; 8(3): e58526.
10. Wandeler G, Keiser O, Mulenga L, et al. Tenofovir in second-line ART in Zambia and South Africa: collaborative analysis of cohort studies. J Acquir Immune Defic Syndr **2012**; 61(1): 41-8.
11. Boender TS, Sigaloff KCE, Hamers RL, et al. Favorable Long-Term Outcomes of 2nd-Line ART Despite Drug-Resistant HIV-1 in Sub-Saharan Africa. Topics in Antiviral Medicine **2014**; 22(e-1).
12. Sigaloff KC, Hamers RL, Wallis CL, et al. Second-line antiretroviral treatment successfully resuppresses drug-resistant HIV-1 after first-line failure: prospective cohort in Sub-Saharan Africa. J Infect Dis **2012**; 205(11): 1739-44.
13. Murphy RA, Sunpath H, Castilla C, et al. Second-line antiretroviral therapy: long-term outcomes in South Africa. J Acquir Immune Defic Syndr **2012**; 61(2): 158-63.
14. Johnston V, Cohen K, Wiesner L, et al. Viral suppression following switch to second-line antiretroviral therapy: associations with nucleoside reverse transcriptase inhibitor resistance and subtherapeutic drug concentrations prior to switch. J Infect Dis **2014**; 209(5): 711-20.
15. Johnston V, Fielding K, Charalambous S, et al. Second-line antiretroviral therapy in a workplace and community-based treatment programme in South Africa: determinants of virological outcome. PLoS One **2012**; 7(5): e36997.
16. Hosseinipour MC, Kumwenda JJ, Weigel R, et al. Second-line treatment in the Malawi antiretroviral programme: high early mortality, but good outcomes in survivors, despite extensive drug resistance at baseline. HIV Med **2010**; 11(8): 510-8.
17. Castelnuovo B, John L, Lutwama F, et al. Three-year outcome data of second-line antiretroviral therapy in Ugandan adults: good virological response but high rate of toxicity. J Int Assoc Physicians AIDS Care (Chic) **2009**; 8(1): 52-9.

18. Adetunji AA, Achenbach C, Feinglass J, et al. Optimizing treatment switch for virologic failure during first-line antiretroviral therapy in resource-limited settings. J Int Assoc Provid AIDS Care **2013**; 12(4): 236-40.
19. Paton NI, Kityo C, Thompson J, et al. Nucleoside reverse-transcriptase inhibitor cross-resistance and outcomes from second-line antiretroviral therapy in the public health approach: an observational analysis within the randomised, open-label, EARNEST trial. Lancet HIV **2017**.
20. Boyd MA, Moore CL, Molina JM, et al. Baseline HIV-1 resistance, virological outcomes, and emergent resistance in the SECOND-LINE trial: an exploratory analysis. Lancet HIV **2015**; 2(2): e42-51.
21. Hosseinipour MC, van Oosterhout JJ, Weigel R, et al. The public health approach to identify antiretroviral therapy failure: high-level nucleoside reverse transcriptase inhibitor resistance among Malawians failing first-line antiretroviral therapy. Aids **2009**; 23(9): 1127-34.
22. Economics IoH. Development of a quality appraisal tool for case series studies using a modified Delphi technique. Alberta, Canada: Institute of Health Economics, **2012**.
23. Liu TF, Shafer RW. Web resources for HIV type 1 genotypic-resistance test interpretation. Clin Infect Dis **2006**; 42(11): 1608-18.
